# Comparison of laboratory and immune characteristics of the initial and second phase of tick-borne encephalitis

**DOI:** 10.1080/22221751.2022.2086070

**Published:** 2022-06-15

**Authors:** Petra Bogovič, Andrej Kastrin, Stanka Lotrič-Furlan, Katarina Ogrinc, Tatjana Avšič Županc, Miša Korva, Nataša Knap, Katarina Resman Rus, Klemen Strle, Franc Strle

**Affiliations:** a Department of Infectious Diseases, University Medical Centre Ljubljana, Ljubljana, Slovenia; b Institute for Biostatistics and Medical Informatics, Faculty of Medicine, University of Ljubljana, Ljubljana, Slovenia; c Institute of Microbiology and Immunology, Faculty of Medicine, University of Ljubljana, Ljubljana, Slovenia; d Laboratory of Microbial Pathogenesis and Immunology, Division of Infectious Diseases, Wadsworth Center, New York State Department of Health, Albany, NY, USA

**Keywords:** Tick-borne encephalitis, tick-borne encephalitis virus, immune mediators, cytokines, chemokines, laboratory findings, liver involvement, initial phase

## Abstract

Tick-borne encephalitis (TBE) usually has a biphasic course which begins with unspecific febrile illness, followed by central nervous system involvement. Because TBE is not yet suspected during the initial phase, knowledge of early TBE pathogenesis is incomplete. Herein we evaluated laboratory and immune findings in the initial and second (meningoencephalitic) phase of TBE in 88 well-defined adult patients. Comparison of nine laboratory blood parameters in both phases of TBE revealed that laboratory abnormalities, consisting of low leukocyte and platelet counts and increased liver enzymes levels, were predominately associated with the initial phase of TBE and resolved thereafter. Assessment of 29 immune mediators in serum during the initial phase, and in serum and cerebrospinal fluid (CSF) during the second phase of TBE revealed highly distinct clustering patterns among the three groups. In the initial phase of TBE, the primary finding in serum was a rather heterogeneous immune response involving innate (CXCL11), B cell (CXCL13, BAFF), and T cell mediators (IL-27 and IL-4). During the second phase of TBE, growth factors associated with angiogenesis (GRO-α and VEGF-A) were the predominant characteristic in serum, whereas innate and Th1 mediators were the defining feature of immune responses in CSF. These findings imply that distinct immune processes play a role in the pathophysiology of different phases of TBE and in different compartments.

## Introduction

Tick-borne encephalitis virus (TBEV), the causative agent of tick-borne encephalitis (TBE), is the most common vector-transmitted viral agent of the central nervous system (CNS) inflammation in the Northern Hemisphere. TBE is caused by three main subtypes of TBEV: European, Siberian, and Far Eastern; recently Baikal and Himalayan TBEV subtypes have also been reported. Differences in the clinical presentation of infection with different virus subtypes have been noted [1,2]. In the majority of patients infected with the European subtype of TBEV, CNS inflammation is preceded by a febrile illness, resulting in a biphasic course of the disease. The initial illness (first phase) which corresponds to viremia, presents with fever, fatigue, malaise, headache, and muscle and joint pain which occur in the absence of CNS inflammation. The initial phase usually lasts less than one week [3–5] and is followed by improvement lasting several days. The hallmark of the second phase of TBE is CNS involvement. Meningitis is the predominant manifestation in children whereas only approximately 50% of adult patients develop meningitis, 40% meningoencephalitis, and 5–10% meningoencephalomyelitis [2,3]. TBE is on average more severe than inflammation of the CNS caused by other viruses. The fatality rate in patients infected with the European TBEV subtype is up to 1%. Moreover, 5% of patients have permanent pareses and more than 30% suffer from postencephalic syndrome [2,4,6–9].

The biphasic nature of TBE and the transient presence of TBEV in blood prove challenging for the diagnosis of this condition. In clinical practice, TBEV infection is detected by serological methods. IgM antibodies typically appear at the end of the first phase of the disease, followed by seroconversion to IgG. However, most patients do not develop detectable levels of IgM and IgG antibodies to TBEV in blood until the beginning of the meningoencephalitic phase of the disease. This delay presents a challenge for early diagnosis, since most patients are still seronegative during the first phase of the illness. TBEV can also be detected directly. However, isolation of the virus from clinical specimens is a time-consuming and technically demanding method that yields limited sensitivity and is feasible only in specially equipped BSL3 laboratories. It is, therefore, not suitable for routine TBE diagnosis. An alternate approach for direct detection is real-time reverse transcription-polymerase chain reaction (RT-PCR) which is highly sensitive, specific, and rapid. However, due to the transient presence of TBEV in blood, the usefulness of RT-PCR in the clinic is restricted to the first phase of TBE. This phase of the disease manifests as an unspecific febrile illness that is clinically difficult to distinguish from other similar illnesses occurring during warm months and is often missed at diagnosis. In practice, TBE is clinically appreciated only after the appearance of the second, meningoencephalitic phase of illness [10,11].

Although the meningoencephalitic phase of the disease is well described [2,4,7,12,13], data on immune and laboratory findings in patients during the initial phase of the TBE are limited. Consequently, comparisons between the two phases are restricted to general observations rather than detailed longitudinal clinical and laboratory evaluation of individual patients. Moreover, differences in the host immune response between the two phases of the diseases have not been assessed.

In this study, we assessed laboratory and immune findings in a well-defined cohort of TBE patients followed longitudinally from initial viremia through CNS involvement during the second phase of TBE. The goal of this work was to inform disease pathogenesis and to identify potential new diagnostic markers for TBE.

## Patients and methods

### Patients

Patients with febrile illness in whom TBEV RNA was detected in the blood during the initial phase and who later developed CNS involvement were enrolled in this study. They were selected from a large pool of adult patients examined for febrile illness following a tick bite or tick exposure at the Department of Infectious Diseases, University Medical Center Ljubljana (Slovenia), during 2003–2019.

The initial phase of TBE was defined as the presence of fever and constitutional symptoms, demonstration of viral RNA in serum or blood, and the absence of signs/symptoms of CNS involvement at the time of actual illness, followed by symptoms and signs of CNS involvement that fulfilled criteria for TBE.

TBE was defined as the presence of clinical signs or symptoms of meningitis or meningoencephalitis, with elevated cerebrospinal fluid (CSF) leukocyte counts (>5 × 10^6^ cells/L), and demonstration of a recent infection with TBEV indicated by serum IgM and IgG antibodies or IgG seroconversion in paired serum samples.

### Ethics

The study was conducted in accordance with the principles of the Declaration of Helsinki, the Oviedo Convention for the Protection of Human Rights and Dignity of the Human Being with regard to the Application of Biology and Medicine, and the Slovene Code of Medical Deontology. The study was approved by the National Medical Ethics Committee of Slovenia (numbers 218/05/09, 178/02/13, 131/06/13, 152/06/13, and 37/12/13). Patients whose specimens were obtained in the study on the aetiology of febrile illness after a tick bite/exposure to ticks signed an informed consent form while the Ethics Committee waived the need for written informed consent for patients for whom remnants of routinely collected serum specimens were used.

### Laboratory testing

Clinical features and laboratory findings were obtained through organized prospective follow-up. C-reactive protein (CRP) concentration, leukocyte count (total and separately neutrophils, lymphocytes, and monocytes), thrombocyte count and aspartate aminotransferase (AST), alanine aminotransferase (ALT), and gamma-glutamyl transferase (GGT) concentrations in serum were determined routinely at the initial examination during the first phase of illness and at the initial examination during the second phase of TBE.

Total RNA was extracted from serum or blood using the QIAamp Viral RNA Mini Kit (QIAGEN, Hilden, Germany) in accordance with the manufacturer’s instructions. For quantitative real-time RT-PCR the TaqMan Fast Virus 1-Step Master Mix (Applied Biosystems, Carlsbad, CA, USA) was used as reported previously [14].

The presence of TBEV antibodies in serum samples was determined using the Enzygnost Anti-TBE/FSME Virus (IgM, IgG) test (Siemens AG, Munich, Germany) in accordance with the manufacturer’s instructions.

**Figure 1.**
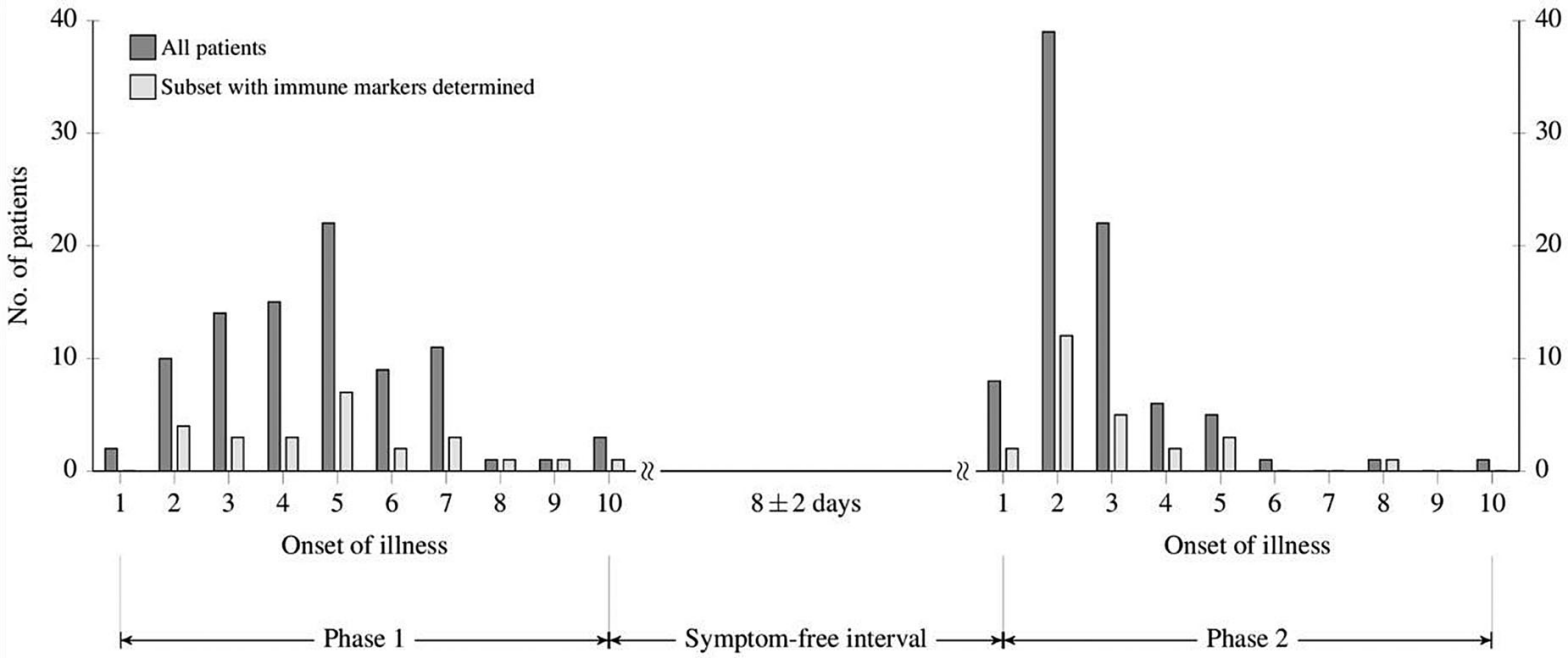
The number of patients included in the study according to the day after the onset of illness when serum specimens in the first phase of TBE, and serum and CSF specimens in the second phase of TBE were obtained.

Specimens for laboratory and immune testing were obtained concurrently and assessed immediately or stored at −80°C for later evaluation (e.g. immune determinations). Figure 1 shows days of illness when specimens were collected in the initial phase (serum) and in the meningoencephalitic phase (serum and CSF) of the disease.

### Immune determinations

Immune responses were assessed in a subgroup of 25 patients. Serum samples were obtained in the initial and second phase of TBE, CSF specimens in the second phase of the disease. All samples had been stored at −80°C until processing. The levels of cytokines and chemokines representing innate (GM-CSF, IFN-α, GRO-α, IL-10, IL-15, IL-1RA, IL-1β, IL-6, IL-8, MCP-1, MIP-1α, TNF-α, VEGF-A, CXCL11, CCL19, IFN-β), adaptive-Th1 (IFN-γ, IL-12p40, IL-2, CXCL10, CXCL9, IL-28B), adaptive-Th2 (IL-4, IL-5), adaptive-Th17 (IL-17A, IL-17F, IL-21, IL-22, IL-23, IL-17E, IL-27), and B cell (CXCL13, CXCL12, BAFF, APRIL) responses were determined using Human Cytokine/Chemokine Magnetic Bead Panel, Human Th17 Magnetic Bead Panel, Human Cytokine/Chemokine Panel II, Human Cytokine/Chemokine Magnetic Bead Panel III and Human Cytokine/Chemokine Magnetic Bead Panel IV (Millipore, Germany), and APRIL Human ProcartaPlex Simplex Kit (ThermoFisher Scientific, Waltham, MA, USA) on a MAGPIX instrument (Luminex, Austin, TX, USA). To minimize interassay variation, all measurements in a single panel were performed on the same day in one complete experiment according to the manufacturer’s instructions. All samples were previously aliquoted and diluted to a final concentration 1:5 with Assay Buffer (Millipore, Germany). For all plates in each panel, simultaneous analysis was done with Milliplex Analyst 5.1 software.

Of the 35 cytokines/chemokines measured, six immune mediators for which no or minimal variability was found (IL-12P40, IL-28B, IL-5, IL-17F, IL-22, and IL-17E) were excluded.

### Statistical analysis

Continuous variables were summarized as median values and interquartile ranges (IQRs); discrete variables were reported as counts and percentages with 95% confidence intervals (CIs). Basic statistical comparisons between TBE phases were based on the Wilcoxon rank-sum test (Table 1) and its pairwise extension (Table 2). The Benjamini–Hochberg procedure to control for false discovery rate in multiple comparisons was used as appropriate.

**Table 1.** Comparison of laboratory findings in patients with the initial and the second (meningoencephalitic) phase of tick-borne encephalitis.

Parameter	Initial phase^a^	Second phase^b^	*P* value
Leukocytes (× 10^9^/L)	2.30 (1.20)	9.30 (3.80)	<0.001
<4 × 10^9^/L	76/86 (88.4, 79.7–94.3)	0/87 (0, 0–4.2)	<0.001
Neutrophils (× 10^9^/L)	1.17 (0.81)	7.00 (3.09)	<0.001
<1.5 × 10^9^/L	49/74 (66.2, 54.3–76.8)	0/86 (0, 0–4.2)	<0.001
Lymphocytes (× 10^9^/L)	0.80 (0.50)	1.50 (0.80)	<0.001
<1.1 × 10^9^/L	56/74 (75.7, 64.3–84.9)	0/85 (0, 0–4.3)	<0.001
Monocytes (× 10^9^/L)	0.28 (0.20)	0.70 (0.40)	<0.001
<0.21 × 10^9^/L	32/74 (43.2, 31.8–55.3)	1/85 (1.2, 0–6.4)	<0.001
Thrombocytes (× 10^9^/L)	132 (49.0)	249 (108)	<0.001
<150 × 10^9^/L	55/86 (64.0, 52.9–74.0)	5/87 (5.7, 1.9–5.9)	<0.001
CRP (mg/L)	5.00 (2.00)	5.00 (4.00)	0.006
≥5 mg/L	13/86 (15.1, 8.3–24.5)	28/86 (32.6, 22.8–43.5)	0.012
AST (µkat/L)	0.60 (0.36)	0.33 (0.14)	<0.001
≥0.53 µkat/L	42/70 (0.60, 47.6–71.5)	13/73 (17.8, 9.8–28.5)	<0.001
ALT (µkat/L)	0.52 (0.39)	0.48 (0.34)	0.583
≥0.58 µkat/L	28/70 (0.40, 28.5–52.4)	25/73 (34.2, 23.5–46.3)	0.590
GGT (µkat/L)	0.36 (0.30)	0.57 (0.52)	<0.001
≥0.64 µkat/L	16/69 (23.2, 13.9–34.9)	31/73 (42.5, 31.0–54.6)	0.024

Data are given as median (interquartile range) or proportion (%, 95% confidence interval). CRP, C-reactive protein; AST, aspartate aminotransferase; ALT, alanine aminotransferase; GGT, gamma-glutamyl transferase.

^a^ Median duration of illness 5 days, range 1–10 days.

^b^ Median duration of meningoencephalitic phase of illness 2 days, range 1–10. Median symptom-free interval 8 days.

**Table 2.** Comparison of levels of cytokines/chemokines in serum and cerebrospinal fluid in 25 patients with the initial and second (meningoencephalitic) phase of tick-borne encephalitis.

Immune response	Cytokine/chemokine	Serum initial phase ng/ml	Serum second phase ng/ml	CSF second phase ng/ml	*P*_1_ value	*P*_2_ value	*P*_3_ value
Innate	GM-CSF	1.79 (1.55)	1.03 (1.46)	1.28 (0.49)	0.152	0.068	0.824
	IFN-α	27.5 (28.5)	11.0 (17.7)	32.3 (19.9)	0.013	0.765	0.013
	GRO-α	242 (155)	366 (273)	0.30 (0.00)	<0.001	<0.001	<0.001
	IL-10	0.12 (0.31)	0.12 (0.00)	0.12 (0.00)	0.152	0.791	0.337
	IL-15	0.32 (0.00)	0.32 (0.00)	0.32 (0.86)	0.744	0.244	0.068
	IL-1RA	0.64 (4.00)	2.27 (5.99)	0.16 (0.00)	0.891	0.191	0.174
	IL-1β	0.37 (0.09)	0.37 (0.30)	0.37 (0.00)	0.559	0.457	0.419
	IL-6	0.23 (0.00)	0.23 (0.00)	54.46 (72.39)	0.583	0.002	<0.001
	IL-8	2.64 (2.89)	3.00 (6.76)	25.39 (22.66)	0.560	0.001	0.024
	MCP-1	300.91 (267.49)	78.67 (41.76)	383.97 (921.00)	<0.001	0.058	<0.001
	MIP-1α	0.36 (0.00)	0.36 (9.87)	0.36 (0.00)	0.259	>0.999	0.029
	TNF-α	8.05 (4.12)	9.16 (16.0)	1.65 (2.01)	0.457	<0.001	<0.001
	VEGF-A	38.5 (20.1)	52.2 (39.8)	25.6 (24.3)	0.003	0.054	0.002
	CXCL11	142 (175)	25.2 (19.3)	4.51 (7.56)	<0.001	<0.001	0.001
	CCL19	62.05 (45.69)	9.89 (16.4)	140 (164)	<0.001	0.002	<0.001
	IFN-β	33.8 (0.00)	33.8 (0.00)	33.8 (0.00)	0.713	0.628	0.824
Th1	IFN-γ	1.31 (1.04)	0.62 (1.02)	4.77 (5.86)	0.029	0.001	0.001
	IL-2	0.33 (0.20)	0.33 (0.02)	0.33 (0.02)	>0.999	0.591	0.545
	CXCL10	1030 (877)	80.8 (21.5)	3325 (5045)	<0.001	0.001	<0.001
	CXCL9	193 (156)	82.6 (58.5)	92.8 (166)	0.001	0.182	0.131
Th2	IL-4	80.64 (171.99)	52.43 (132.49)	0.31 (0.00)	>0.999	0.001	<0.001
Th17	IL-17A	0.58 (0.46)	0.47 (0.35)	0.70 (0.12)	>0.999	0.791	0.821
	IL-21	2.72 (3.95)	5.50 (8.00)	2.72 (0.00)	0.941	0.019	0.002
	IL-23	0.08 (0.92)	0.09 (0.66)	0.08 (0.00)	0.537	0.019	0.010
	IL-27	0.23 (0.17)	0.21 (0.24)	0.04 (0.05)	0.457	0.002	0.002
B cell	CXCL13	14.61 (5.24)	11.63 (5.65)	0.27 (0.00)	0.023	0.002	0.001
** **	CXCL12	724 (204)	778 (196)	602 (357)	0.655	0.591	0.545
** **	BAFF	0.58 (0.56)	0.18 (0.48)	0.06 (0.00)	0.001	0.001	0.001
** **	APRIL	278 (234)	301 (162)	390 (300)	>0.999	0.054	0.046

CSF, cerebrospinal fluid; P_1_, comparison of findings in serum obtained in the initial and in the second phase of tick-borne encephalitis (TBE); P_2_, comparison of findings in serum obtained in the initial phase and CSF obtained in the second phase of TBE; P_3_, comparison of findings in serum obtained in the second phase and CSF obtained in the second phase of TBE.

Exploratory modelling of the dataset was performed using sparse partial least squares regression combined with discriminant analysis (sPLSDA) [15]. The main objective of the sPLSDA method is to (i) transform a large set of observed variables into a low-dimensional matrix of latent components and (ii) induce a classifier to discriminate between target classes (i.e. groups). In this analysis, all variables in the sPLSDA model were centred to a zero mean and scaled to a unit length. The optimal number of latent components was determined by repeated 5-fold cross-validation. R software was used for all statistical analyzes [16].

## Results

Eighty-eight adult patients with biphasic course of TBE, 46 (52.3%) women and 42 (47.7%) men, with median age 49 (range 19–84, IQR 37–61) years, were included in the study. In all 88 patients, the presence of TBEV RNA was established during the initial phase of illness, all the patients later developed CNS inflammation and all seroconverted.

**Figure 2.**
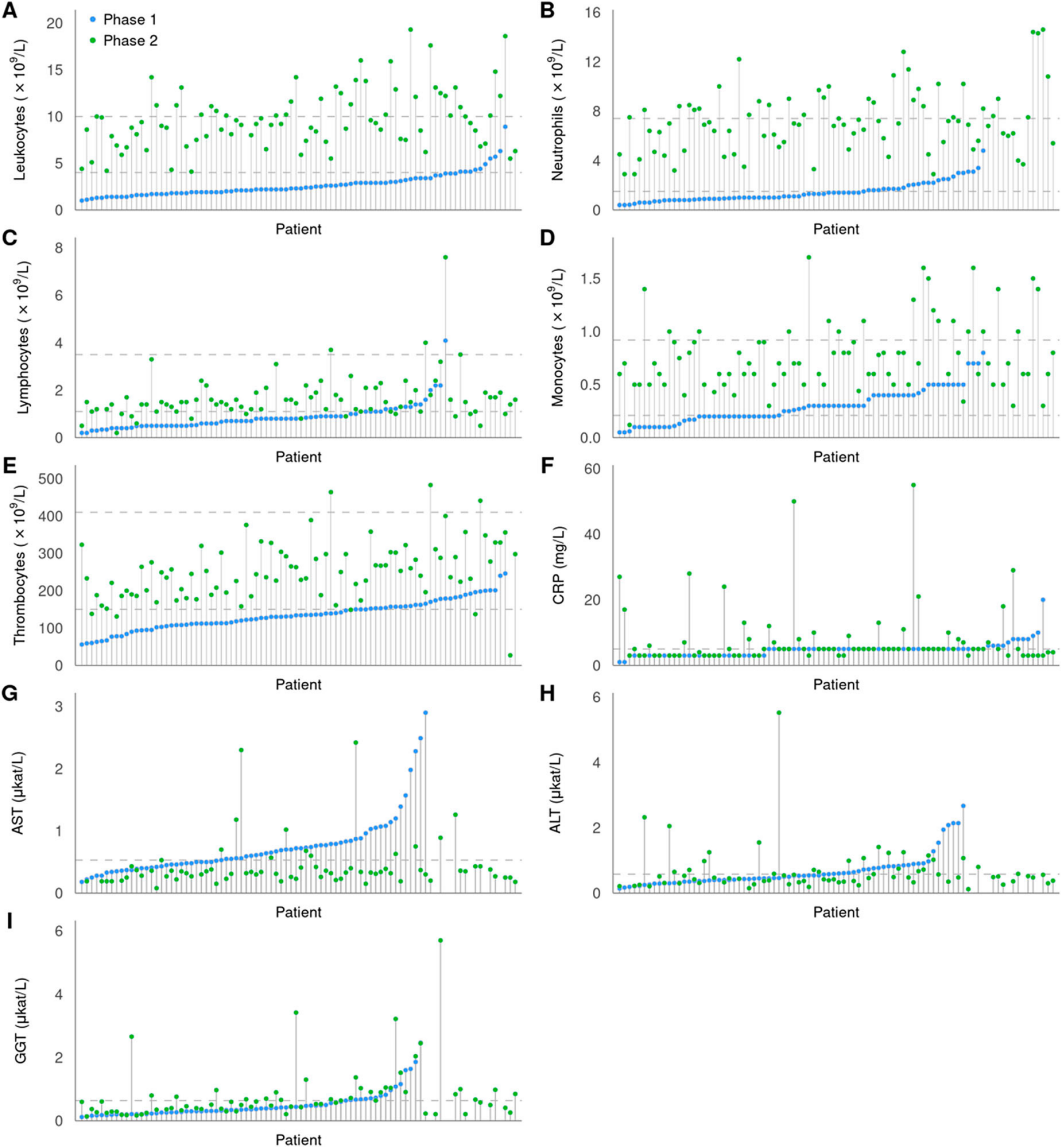
Laboratory results for each individual patient in the first (blue circles) and the second phase of TBE (green circles) in the ascending order according to the findings in the initial phase of illness. Values for patients for whom only results in the second phase of the disease were available are shown on the far right of the images. Dashed lines represent normal ranges for white blood cell counts and thrombocytes, and upper normal level for CRP and liver enzymes.

Comparison of laboratory findings in the initial and the second (meningoencephalitic) phase of TBE revealed significant differences in peripheral blood leukocyte counts (including neutrophil, lymphocyte, and monocyte counts) and platelet counts, as well as serum concentrations of CRP, AST, and GGT but not for ALT (Table 1). Direct comparison of these laboratory results between the initial and meningoencephalitic phase for individual patients is shown in Figure 2.

### Leukocyte and platelet counts

Total peripheral blood leukocyte counts were diminished (<4.0 × 10^9^/L) in 88.4% of patients (76/86), during the initial phase of illness, with the lowest value 1.0 × 10^9^/L. In all patients, leukocyte counts returned to normal (4.0 × 10^9^/L to 10.0 × 10^9^/L; 59.8%) or became elevated (>10.0 × 10^9^/L; 40.2%) in the second phase, with the highest value of 19.3 × 10^9^ cells/L. The increase in the second phase of illness was seen not only in patients with leukopoenia but also in those who had leukocyte count in the normal range during the initial phase of TBE. Analogous dynamics were seen in all subgroups of leukocytes, but were most pronounced for neutrophils and slightly less marked for monocytes and lymphocytes. Similar pattern was also observed for platelet counts; in the initial phase 55/86 (64.0%) patients had thrombocytopenia (<150 × 10^9^/L) with a shift to normal range in the meningoencephalitic phase in all but 5 (6%) patients (Table 1, Figure 2).

### Liver enzymes, CRP concentration

In the initial phase of TBE, patients often had elevated levels of serum AST (42/70, 60%) or ALT (28/70, 40%). These markers were generally mildly elevated, but several patients had impressive increase in AST and/or ALT levels (Figure 2). No obvious explanations for these prominent elevations were noted, and the levels returned to the normal range during the meningoencephalitic phase of TBE (i.e. within 10 days). Although for both tested parameters there was a tendency for lower (normal) levels during the meningoencephalitic phase of illness, the differences were significant only for AST. However, several patients with the levels of AST or ALT within the normal range during the initial phase of illness developed elevated levels of the markers during meningoencephalitic phase of illness for which work-up revealed no reliable explanation and which normalized during later follow-up. There were also significant differences between GGT levels in the initial and second phase of TBE, however, the dynamics were opposite than for AST and ALT: the levels and the proportion of patients with elevated GGT values were significantly lower in the initial than in the second phase of TBE. Similar pattern was found also for serum CRP concentrations.

### Immune mediators

**Figure 3.**
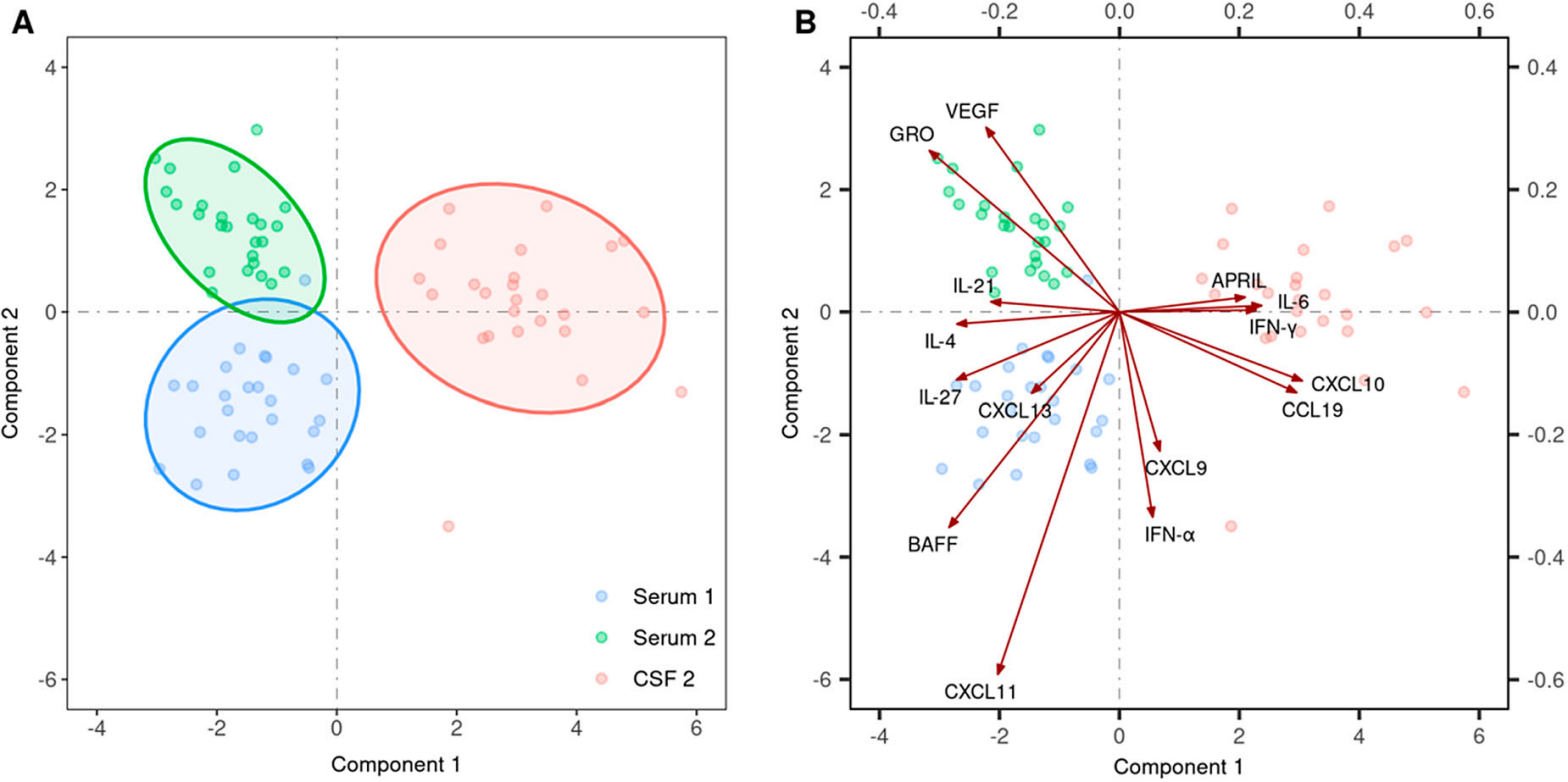
Comparison of the levels of 29 cytokines/chemokines in 25 patients with biphasic course of TBE performed by exploratory modelling using sparse partial least squares regression combined with discriminant analysis (sPLSDA). (A) shows an individual plot of sparse PLS-DA with the first two PLS components of immune mediators in serum of the initial phase of TBE (blue dots, Serum 1), in serum of the second phase of the disease (green dots, Serum 2), and in CSF of the second (meningoencephalitic) phase of TBE (red dots, CSF 2). The biplot in (B) extends the left panel by considering the contributions of the 15 most discriminative mediators between the three groups, with the length of the arrows corresponding to the importance of the mediator.

To evaluate the status of host immune responses, the levels of 29 cytokines and chemokines associated with innate and adaptive (T and B cell) responses were assessed in serum and CSF in the initial and the second (meningoencephalitic) phase of TBE in 25 patients. Several statistically significant differences were found in serum between the initial and second phase of TBE as well as between serum and CSF during the second (meningoencephalitic) phase of TBE. Exploratory modelling using sPLSDA demonstrated distinct clustering of the immune mediators in the three groups (serum of the initial phase of TBE, and serum and CSF in the second phase of the disease) (Figure 3(A)). These findings imply differences in immune responses according to the temporal evolution of the disease and between different compartments (serum or CSF) (Table 2, Figure 3). The key immune mediators that discriminate between the three groups are depicted in Figure 3(B). The primary feature during the initial phase of TBE in serum was a rather heterogeneous response involving innate (CXCL11), B cell (CXCL13, BAFF), and T cell mediators (IL-27 and IL-4) whereas growth factors associated with angiogenesis (GRO-α and VEGF-A) were the most discriminative feature of serum responses in the second phase of TBE. In contrast, innate (IL-6), Th1 mediators (IFN-γ, CXCL10, CCL19), and B cell mediator APRIL were a characteristic feature of immune responses in CSF, the site of the disease during the meningoencephalitic phase of TBE (Table 2, Figure 3(B)).

## Discussion

Knowledge of TBE is limited, particularly during the first phase of the disease which is as a rule clinically appreciated as a manifestation of TBEV infection only in retrospect; after the appearance of CNS involvement. Thus, comparisons of results in the same patients in the first and second phase of the disease are confined predominantly to small cohorts and the information is limited to basic laboratory findings such as total blood leukocyte and platelet counts. Although the host immune responses are thought to play an important role in the infection and presumably the pathogenesis of the disease [12,13,17,18], information on immune mediators during the initial phase of TBE is absent. The goal of our study was to assess in greater detail laboratory and immune mediators in a relatively large cohort of patients during the initial phase of TBE and the second phase of this disease. This was made possible because of our large collection of clinical samples from TBE patients seen at our centre over a 20-year period.

A key finding of this comparison was that 8 of the 9 laboratory parameters assessed differed significantly between the first and second phase of the disease. Seven parameter abnormalities were associated with the initial phase of TBE, including: total serum leukocyte (including each of leukocytes subsets) and platelet counts were reduced whereas AST and ALT values were elevated. In contrast, these parameters normalized in most patients in the second phase (Table 1). These findings corroborate a temporal association of the abnormalities with the presence of TBEV in the blood and suggest impairment of the bone marrow as well as liver by systemic TBEV infection. These systemic responses are abrogated in the second phase of TBE during which the disease (and the infection) is thought to be localized to the CNS. The mechanisms of the damage may be related directly to the presence of TBEV or indirectly to the host immune response, however, specific underlying mechanisms have not been elucidated. Nevertheless, since the proportion of patients with abnormalities varied greatly for individual parameters (from 40% for monocytopenia to 88% for leukopoenia) and since the levels of GGT and CRP were more often abnormal in the second phase whereas other parameters were abnormal in the initial phase of TBE, the pathogenic mechanisms leading to abnormalities are likely not uniform (the same). These general laboratory patterns are further substantiated by findings from individual patients. In the initial phase of TBE, total leukocyte, neutrophil, lymphocyte, monocyte, and platelet counts were reduced in the majority of patients and had a rather continuous (homogeneous) distribution. These parameters increased in the second phase of illness in a large majority of patients, including those who had counts in the normal range during the initial phase of TBE. In contrast, abnormalities of liver enzymes were somewhat less frequent and relatively mild in most patients but in a few cases, they were rather pronounced; this “dispersed” pattern was seen not only during the initial phase of TBE but also during the meningoencephalitic phase, and such elevations were generally not seen in the same patient in both phases of the disease. Since patients with TBE as a rule have headaches and fever, a reasonable explanation for abnormal liver test results could have been liver tissue damage associated with the usage of antipyretics and/or analgetics. However, a large majority of our patients were not receiving any drug at the time of obtaining blood and CSF specimens and a few days earlier.

While the majority of laboratory findings of the present study is of clinical relevance, this is not valid for CRP. Although the levels and the proportion of patients with elevated serum concentrations of CRP were significantly higher in the meningoencephalitic phase than in the initial phase of TBE, in the greater part of patients the levels were in the normal range or only mildly elevated. Consequently, the significance of CRP levels for distinguishing between the first and second phase of TBE is clinically negligible. However, CRP levels may be important for distinguishing between the first phase of TBE and some other febrile illness such as human granulocytic anaplasmosis (HGA). HGA is a bacterial disease caused by *Anaplasma phagocytophilum* and, like TBEV, is transmitted by ticks [19,20]. The clinical presentation of HGA is very similar to the first phase of TBE: both are febrile illness with non-specific symptoms, and are often accompanied by laboratory abnormalities such as leukopoenia, thrombocytopenia and elevated liver test results. However, HGA is associated with laboratory signs of inflammation such as robustly elevated CRP levels while in the initial phase of TBE CRP levels are as a rule in the normal range. The distinction between the two diseases is of importance because proper diagnosis enables etiologic treatment of HGA, a potentially fatal disease, with antibiotics while for TBE no etiologic treatment exists [19,20].

Reports on the immune markers in patients with TBE were often limited to a few immune markers and were seldom accompanied with well-defined clinical information making it difficult to link the immune findings to disease presentation. Moreover, most studies did not evaluate both serum and CSF compartments or the duration of illness. Finally, in the large majority of studies, immune mediators were assessed solely during the meningoencephalitic phase of TBE [12,13,21–37], in only a few also later after acute illness [18,24] but none of the published studies included information on the immune mediators in the initial phase of TBE.

A review of the published information revealed that several cytokines are elevated during the second phase of TBE, including IL-1α, IL-2, IL-6, IL-8, IL-12, IL-15, IFN-α, IFN-γ, and TNF, and growth factors, such as hepatocyte growth factor and vascular endothelial growth factor in sera [12,21,27,30]. In CSF, increased levels of CXCL9 and CXCL10, neutrophil chemoattractants CXCL1 and CXCL2, as well as CCL5 (RANTES), CXCL11, CXCL12, CXCL13, and CCL3 have been reported [22,25,29,33]. Furthermore, in five studies, concentrations of inflammatory mediators were associated with disease severity or unfavourable outcome of the disease [13,18,24,26,38]. Elevated CSF levels of IFN-γ, IL-4, IL-6, and IL-8 in children were associated with the development of sequelae after TBE [31]. In adults, low levels of IL-10 in the CSF later during the second phase of TBE (day 7–18) correlated with a more severe disease [24]. In the other study, positive correlations were found between greater disease severity and higher CSF levels of IL-1, CCL3, IL-12P40, and IL-12P70, and serum levels of CXCL9 and CXCL10 [13]. Finally, in a study of adult patients with TBE serum levels of Th17 mediators were lower in patients with unfavourable outcome whereas those associated with other adaptive or innate immune responses were higher at the last visit in those with an unfavourable outcome [18].

Our previous findings demonstrate that during the meningoencephalitic phase of TBE, innate, and Th1 adaptive inflammatory mediators are highly concentrated in CSF, the site of the disease, while inflammatory mediators associated with B cell responses, and particularly Th17 responses appear concentrated in serum [13]. However, since in the previous study we did not evaluate the illness prior to CNS involvement, we could not exclude the possibility that differences between the immune responses in CSF and serum reflect the temporal lag between the events in the two compartments, i.e. that immune responses seen in CSF during CNS involvement had been present in serum in the initial (pre-CNS) phase of the disease. The current study shows that the immune response in serum in the initial phase of TBE is distinct from the responses in serum and CSF in the second, meningoencephalitic phase of the disease (Figure 3(A)) implying that distinct immune processes take place at different phases and different sites. The latter is consistent with previous findings [13] indicating that inflammatory parameters in serum and CSF at the time of CNS inflammation differ. These differences are likely due at least in part to the presence and extent of viremia, i.e. the fact that TBEV is present in blood solely during the initial phase of TBE but not during meningoencephalitic phase of illness, and can only be very exceptionally demonstrated in CSF [39].

To gain insights into the immune events during different phases of TBE, cytokines/chemokines were classified into group according to their main predicted function; innate (including growth factor responses), T cell (Th1, Th2, or Th17), and B cell. Moreover, we used unbiased statistical analyses to ascertain the key immune response patterns in each compartment. These analyses revealed distinct clustering patterns among cytokines and chemokines in serum obtained during the initial phase of illness and those in serum or CSF during CNS involvement. Furthermore, these data also exposed the most significant classifiers associated with each compartment at each phase of the disease. The primary feature in serum during the initial phase of TBE was a rather heterogeneous response involving innate (CXCL11), B cell (CXCL13, BAFF), and T cell mediators (IL-27 and IL-4). In contrast, the immune responses during the CNS phase of the disease, were characterized by growth factors associated with angiogenesis (GRO-α and VEGF-A) in serum, whereas innate (IL-6) and Th1 mediators (IFN-γ, CXCL10, CCL19) were a characteristic feature of immune responses in CSF. GRO-α and VEGF-A are angiogenic factors and are associated with increased permeability of peripheral and CNS vessels which could lead to the disruption of the blood–brain barrier and consequently contribute to the development of encephalitis [12].

The advantage of our study is a unique, clinically and microbiologically well-defined group of 88 patients with a biphasic course of TBE, compounded by precise laboratory data for most patients, and immune indicators for a subset of 25 patients, in the first and second phase of the disease that enabled the direct comparison of findings in the first and second phase of the disease in the same patients. Furthermore, an unbiased statistical approach was employed that revealed distinct clustering patterns among groups and highlighted the most discriminative individual immune mediators for each of the three groups. This information allows a more rational choice of mediators, which would reduce the cost of further research and decrease “noise” due to the large number of results with poor resolution and thus facilitate or improve statistical assessments. One of the limitations of the present study is that paired laboratory results in the first and the second phase of TBE were not available for all patients. Furthermore, since only adult patients were included, the results may not be completely suitable for children. Although this study is mainly descriptive, we hope that these initial insights will encourage analysis of the clinically relevant mechanisms behind the findings.

In conclusion, the initial phase of TBE is associated with transitory laboratory abnormalities suggesting bone marrow as well as liver involvement and with rather heterogeneous response involving innate (CXCL11), B cell (CXCL13, BAFF), and T cell mediators (IL-27 and IL-4), whereas the second phase of TBE is characterized with angiogenic immune responses in serum and marked innate and Th1 adaptive immune responses in CSF implying that distinct processes play a role in the pathophysiology of different phases of TBE.
